# Supplementary material for: Quartz Crystal Microbalance Measurement of Histidine-Rich Glycoprotein and Stanniocalcin-2 Binding to Each Other and to Inflammatory Cells
Source: Cells. 2022 Aug 29;11(17):2684. doi: 10.3390/cells11172684 (PMC9454698; doi:10.3390/cells11172684)
Supplement: Supplementary file 1 [file cells-11-02684-s001.zip › cells-1873203-supplementary.pdf]

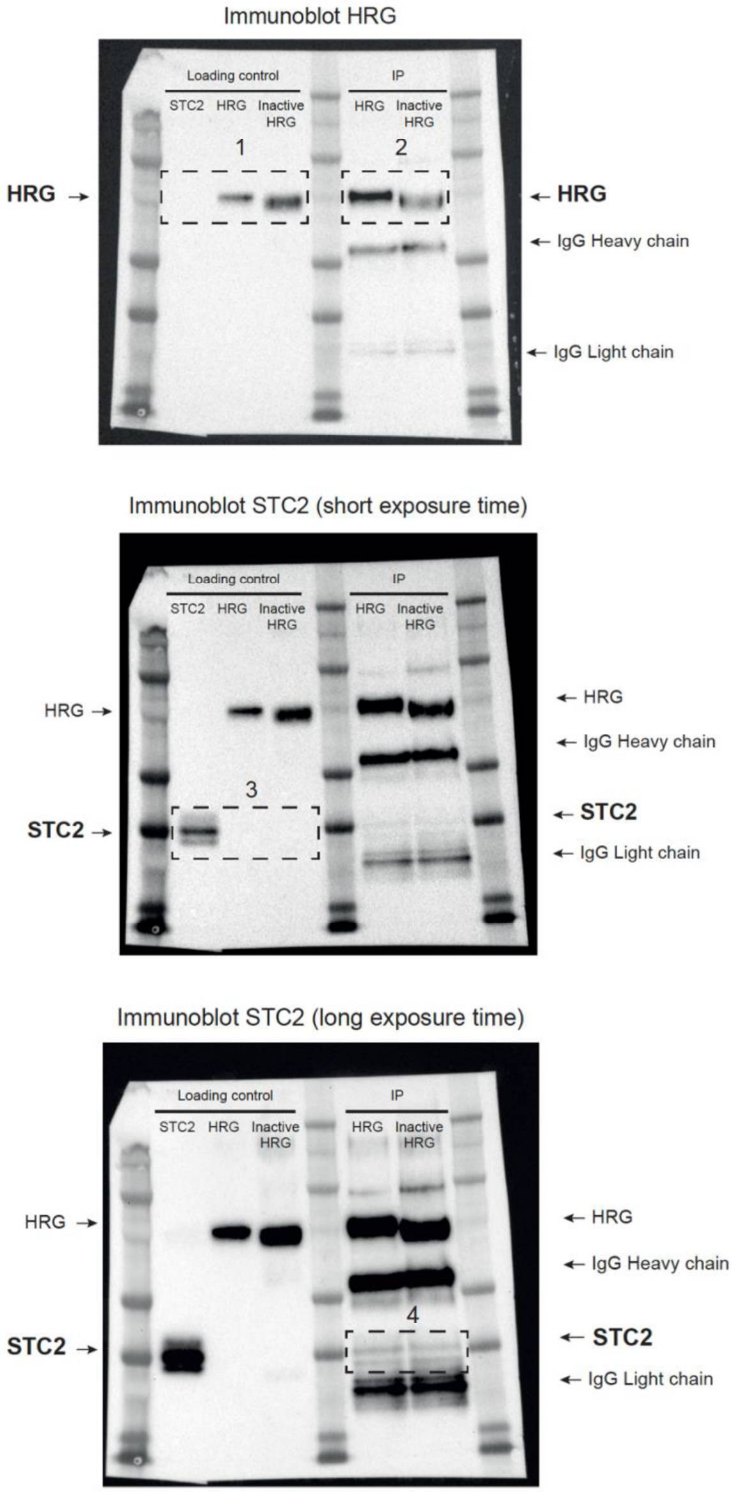

Supplemental Figure S1. Uncropped blots corresponding to Figure 1a

Uncropped immunoblots for HRG (top panel), STC2 (short exposure; middle panel) and STC2 (long exposure, lower panel) were used to compose Figure 1a. Boxes 1-4 in the three blots correspond to parts in the composite in Figure 1a as follows: Box 1; upper left part of the composite, box 2; upper right, box 3; lower left, and box 4; lower right part of the composite, respectively. Migration positions for HRG, STC2 and IgG heavy and light chains are indicated.
